# The Scope of Kidney Affection in Monoclonal Gammopathies at All Levels of Clinical Significance

**DOI:** 10.4274/tjh.2017.0197

**Published:** 2017-12-01

**Authors:** Şadiye Mehtat Ünlü, Hayri Özsan, Sülen Sarıoğlu

**Affiliations:** 1 Dokuz Eylül University Faculty of Medicine, Department of Pathology, İzmir, Turkey; 2 Dokuz Eylül University Faculty of Medicine, Department of Hematology, İzmir, Turkey

**Keywords:** Monoclonal gammopathy of renal significance, Plasma cell disorders, Multiple myeloma, Renal involvement, Kidney, Cast nephropathy

## Abstract

Multiple myeloma (MM) is one of the most important clonal malignant plasma cell disorders and renal involvement is associated with poor prognosis. Although there are several reasons for renal impairment in MM, the main cause is the toxic effects of monoclonal proteins. Although cast nephropathy is the best known and unchallenged diagnosis for hematologists and pathologists, the renal effects of monoclonal gammopathy can be various. Monoclonal gammopathy of renal significance was proposed by the International Kidney and Monoclonal Gammopathy Research Group for renal lesions in monoclonal gammopathy in recent years. Renal lesions in monoclonal gammopathy can be grouped as follows: light chain (cast) nephropathy, acute tubular injury/necrosis, tubulointerstitial nephritis, amyloidosis, monoclonal Ig deposition diseases, immunotactoid glomerulopathy, type I cryoglobulinemia, proliferative glomerulonephritis with monoclonal IgG deposits, C3 glomerulopathy with monoclonal gammopathy, and crystal-storing histiocytosis, considering the previous and new terminology. In this study, renal involvement of monoclonal gammopathies, in terms of previous and new terminology, was reviewed.

## Abstract (tr)

Multipl miyelom (MM) malign plazma hücre hastalıklarının en önemlilerinden biridir ve böbrek tutulumu kötü prognozla ilişkilidir. Multipl miyelomda böbrek fonksiyonunu etkileyecek çeşitli faktörler bulunmakla birlikte ana etken monoklonal proteinlerin toksik etkisidir. Monoklonal gammopatilerde böbrek tutulumları içinde hematolog ve patologlar tarafından en iyi bilinen ve tanısı sorunsuz olan “kast nefropatisi” olmakla birlikte çok farklı tutulum tipleri olabilir. Yakın zamanda “International Kidney and Monoclonal Gammopathy Research Group” tarafından, monoklonal gammopatilerdeki böbrek tutulumlarını bir çatı altında toplayan “renal öneme sahip monoklonal gammopati” terimi önerildi. Monoklonal gammopatilerdeki böbrek lezyonları, önceki ve yeni terminoloji uyarınca; hafif zincir kast nefropati, akut tübüler zedelenme/nekroz, tubulointerstisyel nefrit, amiloidoz, monoklonal Ig depo hastalıkları, immünotaktoid glomerülopati, tip I kriyoglobulinemi, monoklonal IgG depoziti ilişkili proliferatif glomerülonefrit, monoklonal gammopati ilişkili C3 glomerülopati ve kristal/histiyosit depo hastalığı olarak gruplanabilir. Bu makalede, monoklonal gammopatilerdeki böbrek tutulumu yeni ve önceki terminolojiler gözden geçirildi.

## INTRODUCTION

Each specific immunoglobulin (Ig) molecule, which is synthesized by plasma cells, includes two identical heavy chains and two light chains (LCs). There are 5 types of heavy chains (ϒ/α/μ/δ/Є), and immunoglobulins get their names (IgG, IgA, IgM, IgD, IgE) according to these, while there are 2 types of LC (К/λ). Each heavy chain and LC has constant domains and variable domains. Variable domains include the antigen-binding region. Heavy chains and LCs are synthesized independently of each other and finally unite in the endoplasmic reticulum, and the structure called immunoglobulin is unified here [1,2].

Normally, LCs are freely filtered through the glomerular basal membrane because they are low-molecular-weight proteins and are reabsorbed by the proximal tubules, endocytosed and catabolized in the lysosome. After the catabolization process, their amino acids return to the circulation. There are cubilin-megalin receptors on the brush-border of proximal tubular cells, which are very important for the control of LC endocytosis. The heavy chains do not cross the glomerular filtration barrier [3,4,5].

In plasma cell dyscrasia, there is monoclonal plasma cell proliferation and a single type of whole Ig or a subunit or just a LC, which is synthesized by the clone, and the type of these protein fragments causes damage to the kidneys at a varying degree or the amount of the monoclonal LC in the filtrate exceeds the capacity of proximal tubular cells. Approximately 85% of all LCs with plasma cell dyscrasia are nephrotoxic. Most of them are tubulopathic (70%) and so they affect the tubulointerstitial compartment, and the rest of them are glomerulopathic and affect the glomerular compartment. Of course, some host factors are deterministic for this pattern and the grade of the damage, while the molecular and physicochemical characteristics of the LC affecting different compartments are unknown [6].

Monoclonal protein secretion is a typical feature of plasma cell disorders and may affect the kidneys in several ways. Multiple myeloma (MM) is one of the most important clonal malignant plasma cell disorders and renal involvement is associated with poor prognosis [7]. Clonal evaluation steps for MM include monoclonal gammopathy of undetermined significance (MGUS) and smoldering MM [8,9]. While MGUS and smoldering MM patients do not require therapy except for clinical trial settings, if the patients have myeloma defining events such as CRAB (hypercalcemia, renal impairment, anemia, and bone lesions), treatment becomes obligatory. One of the CRAB finding is renal impairment and its incidence ranges from 20% to 50% according to how it is defined [7,9]. The International Myeloma Working Group consensus defines kidney impairment as an acute deterioration of kidney function that results in a serum creatinine level of more than 2.0 mg/dL, but kidney biopsy is currently not proposed. That means that the real incidence of nephropathy is not clear. Although the main cause for renal impairment is the toxic effects of monoclonal proteins, there are several other factors like hypercalcemia, dehydration, nephrotoxic drugs, and contrast agents that can aggravate the underlying disease [9]. Although MGUS and smoldering MM seem to be more benign disorders and do not require therapy, they may cause some renal conditions and those should be treated.

Until recent time, in the writings on this subject, “MM, MGUS, or smoldering myeloma” has probably been the most important classification for hematologists while “glomerulopathic/tubulopathic pattern” has been the most important for pathologists. Although these classifications still maintain their importance, they have become inadequate for the diagnosis, monitoring, and treatment of kidney lesions.

Although MM and cast nephropathy are the most straightforward diagnoses for hematologists and pathologists, there are uncertain areas in this wide clinical and morphologic spectrum for both hematologists and pathologists. According to the treatment guidelines, chemotherapy is indicated when the patient has symptoms related to the underlying plasmacytic or lymphocytic proliferation [10,11]. At this stage, due to the lack of end-stage organ involvement according to the definitions of smoldering MM (>3 g/dL of monoclonal protein and/or >10% bone marrow plasma cells) and MGUS (<3 g/dL of monoclonal protein and/or <10% bone marrow plasma), they do not receive treatment. Although this group looks like benign disease to hematologists, the same situation is not true for the kidneys [11,12,13].

Some patients with proteinuria or acute renal failure consult with nephrologists before hematologists and they get a diagnosis from renal biopsy if the pathologist is experienced in nephropathology, because renal injury patterns other than cast nephropathy and amyloidosis can be very silent or can be confused with other renal diseases.

Until recently, in cases of plasma cell dyscrasia, renal involvements have been grouped into the following categories: LC (cast) nephropathy, acute tubular injury/necrosis (ATD), tubulointerstitial nephritis (TIN), amyloidosis, and monoclonal Ig deposition diseases (MIDDs).

**Figure 1:**
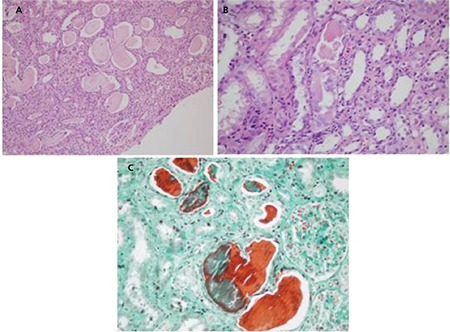
Light chain cast nephropathy: A) classical large myeloma casts show irregular shape and fracture planes (hematoxylin and eosin; 100^x^); B) giant cell surrounding the casts (hematoxylin and eosin; 200^x^); C) metachromatic staining of the myeloma casts (Masson’s trichrome; 200^x^).

Light chain (cast) nephropathy is characterized by acute renal deterioration or clear renal failure and an uncomplicated histopathological diagnosis. Because the formation of the myeloma cast begins in the collecting ducts, the medulla has special importance in the biopsy. The casts generally contain the LCs and Tamm-Horsfall protein and sometimes cellular debris and crystals and giant cells. While monoclonal LC staining is important in immunofluorescence, equally strong staining must also not be ignored (Figure 1) [6,14,15,16].

ATD is especially characterized by proximal tubular damage because the LCs are metabolized in the lysosomes of these cells. ATD can be the only histological lesion in a biopsy or it may be combined with other findings with associated monoclonal gammopathy. The clinical manifestation is a varying degree of renal failure according to the severity of the tubular injuries. Light chain proximal tubulopathy is characterized by periodic acid-Schiff-negative crystals or non-crystallized LCs in the cytoplasm of proximal tubular cells. Pathologic LCs are almost always kappa in proximal tubulopathy with crystal formation and appear to be hypereosinophilic under a light microscope. In addition, round/rectangular free structures can be detected in the cytoplasm of proximal tubules by electron microscope. Due to the loss of crystals during standard frozen sectioning, direct immunofluorescence (DIF) is not a proper method to demonstrate these structures [17,18].

In the non-crystallized cases, pathologic LCs can be kappa or lambda and can be detected in classical DIF. They appear to be hypereosinophilic inclusions in the cytoplasm of proximal tubules in hematoxylin-eosin, dark positive dots in periodic acid-methionine silver, and electron-dense large phagolysosomes by electron microscope. Since the proximal tubule epithelial cells are the physiologic catabolism area for LCs, the acute or chronic signs of tubular injury should be well demonstrated morphologically by the pathologist to differentiate the physiologic process.

**Figure 2:**
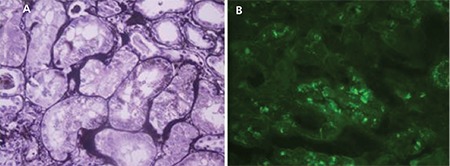
Light chain proximal tubulopathy: A) dark positive dots are a clue for the accumulation of light chain protein in proximal tubules (PAMS; 400^x^); B) cytoplasmic granular staining with direct immunofluorescence for lambda light chain (fluorescein, 400^x^).

Fanconi syndrome, which is characterized by electrolyte wasting and aminoaciduria, is most frequently accompanied by LC proximal tubulopathy with crystals (Figure 2) [19,20].

**Figure 3:**
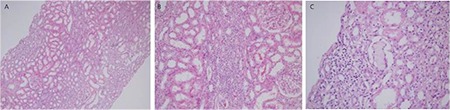
Light chain-related tubulointerstitial nephritis: A-B) interstitial lymphocyte-rich inflammation (hematoxylin and eosin; 100^x^ and 200^x^); C) very few cellular cast formations may be the only clue for monoclonal gammopathy (hematoxylin and eosin; 400^x^).

TIN is quite rare in the plasma cell dyscrasia-associated disease group. Acute renal failure is the most common clinical manifestation. The histomorphological findings are identical to the other inflammatory TIN findings and the morphological diagnosis is very easy; however, it is difficult to establish the connection with plasma cell dyscrasia in terms of etiology and the rate of establishing the linkage between the two diseases is very low. Linear monotypic light staining throughout the tubular basement membranes sometimes can be helpful (Figure 3) [6,21,22,23].

**Figure 4:**
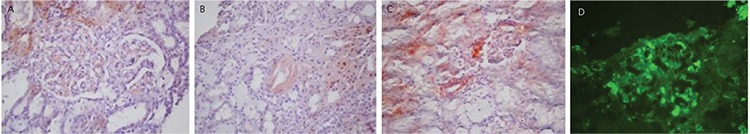
AL amyloidosis. Acellular brick red mesangial deposits with Congo red in the glomerulus (A) and also a vessel wall (B) (400x). C) Yellow-orange birefringence in glomerulus and vessels under polarized light with Congo red (200^x^). D) Mesangial lambda light chain amyloid deposits with direct immunofluorescence (fluorescein, 400^x^).

Amyloidosis is a group of misfolded protein disorders and has the structure of fibrils. Amyloidosis with plasma cell dyscrasia is an important systemic problem. Amyloid light-chain (AL) amyloidosis stains with Congo red just like the amyloid A type and exhibits yellow/orange/green colors under polarized light [24]. Amyloid fibrils consist of generally light (most frequently λ) and sometimes heavy chain fragments. Renal involvement is not infrequent (70%-80%) and it mostly affects the glomeruli and vessels. Although renal involvement is common, cardiac involvement is important for early mortality. Amyloidogenic LCs are directly toxic to the myocardium and rapid suppression is crucial for mortality. It is important to emphasize that amyloidosis patients mostly have a low-grade plasma cell clone and only 20% of patients meet the criteria for MM (Figure 4) [25,26,27,28,29].

**Figure 5:**
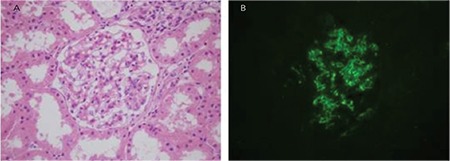
C3 glomerulopathy with monoclonal gammopathy: A) mesangial matrix expansion (hematoxylin and eosin; 400^x^); B) positivity of the mesangial complement C3c with direct immunofluorescence (fluorescein, 400^x^).

MIDDs are systemic like amyloidosis and are observed in approximately 3%-5% of myeloma cases. Although immunoglobulin is stored in many organs, the kidneys are most commonly affected. Except for a few cases, usually the immunoglobulin depositions consist of LCs. Most light-chain deposition disease patients have proteinuria, with 50% of them being in the nephrotic range and about 30% of cases having acute renal failure in the diagnosis. In more than half of the cases, clinical findings meet the criteria for MM. However, it should be kept in mind that a significant number of cases have normal bone marrow biopsy results. Most light microscopic findings are nodular glomerulopathy and monotypic LC deposition in the glomerular-tubular basement membrane and in vessels walls with immunofluorescence (Figure 5) [18,22,30,31,32,33].

A new term, monoclonal gammopathy of renal significance (MGRS), was recently proposed by the International Kidney and Monoclonal Gammopathy Research Group for renal lesions in monoclonal gammopathy. All renal lesions related to monoclonal gammopathy were reclassified under the title of MGRS and it was emphasized that the kidneys of patients whose clinical symptoms do not meet the criteria for MM, Waldenström macroglobulinemia (WM), chronic lymphocytic leukemia (CLL), or other plasma/B-cell proliferative disorders [12] should be monitored (Schema 1). Cast nephropathy and WM (both of which are well recognized for therapy by nephrologists and hematologists) causing acute renal failure are excluded from this description, directly related to high tumor load. Furthermore, as our experience supports, different patterns with different levels can be seen in the same case. If the patient has acute renal failure due to cast nephropathy, hematologists should immediately determine whether the patient has a clinical diagnosis of MM or not, because they know very well that MM is characterized by high tumor mass and high monoclonal Ig level.

However, in MGRS, the patient has a small clone of lymphoplasmacytic cells and the structural and biological features rather than the amounts of immunoglobulin are especially important for renal disease.

Renal dysfunction is a common complication (approximately 20%-25%) in active MM cases and the grade of renal dysfunction is probably correlated with myeloma cell load. Half of patients (50%) even show an improvement in the renal dysfunction with the treatment of myeloma, but the remaining ones have some degree of persistent chronic kidney disease and 2%-12% of these patients need renal transplantation. The high tumor burden and excessive monoclonal protein secretion are the main courses of the renal lesions in this group and the most common manifestation is acute renal failure due to cast nephropathy [34,35]. In MGRS cases, however, tumor burden is not high and the determination of the latent renal injury is based on the physicochemical property of paraprotein [12,36]. As shown in Schema 1, MGRS-associated renal lesions mainly affect the glomerulus.

Fibrillary glomerulonephritis is characterized by fibril structures, resembling amyloidosis. However, these fibrils do not create the amyloid form and do not react with Congo red. Very few are cases associated with monoclonal gammopathy and most cases show a membranoproliferative glomerulonephritis (MPGN) pattern under light microscope and IgG4, IgG1, and polyclonal LC positivity in immunofluorescence. The fibrils are non-branching and randomly oriented, similar to the amyloid fibrils ultrastructurally in electron microscopic assessment, but the fibril diameters are thicker (12-25 nm in diameter) than the amyloid fibrils (8-12 nm in diameter) [37,38,39].

Immunotactoid glomerulopathy is characterized by microtubule organization consisting of monoclonal Ig. More than half of such patients have CLL or a small lymphoma and rarely a low-grade plasma cell clone. In renal biopsy, membranous nephropathy and the MPGN pattern are mostly seen by light microscope and generally monocytic IgG, C3, and LC positivity are seen in immunofluorescence. Similarly to other fibrillary lesions, electron microscopic assessment is very critical for the diagnosis [36,37,40,41].

Type I cryoglobulinemia arises from monoclonal immunoglobulin. In several diseases in which the entire Ig is secreted, such as MGUS, WM, or other B-cell lymphoid disorders, the cryoglobulins can be detected. Renal involvement is more common in the IgG type of cryoglobulinemia and about 30% of patients are affected. Despite episodes of acute renal failure or nephrotic syndrome, the essential process is a chronic glomerular injury. Endocapillary glomerulonephritis or MPGN patterns are the most common morphological findings with the hyaline-protein thrombi in the glomerulus. Monoclonal heavy chains and LCs (most frequently IgG-k) with complements (C3/C4/c1q) are detected in immunofluorescence [42,43,44].

Proliferative glomerulonephritis with monoclonal IgG deposits is a new entity that shows non-organized glomerular deposits most commonly consisting of IgG3-k. Membranoproliferative/endocapillary proliferative and atypical membranous are the most commonly observed patterns by light microscope. Unlike in other deposit diseases (Randall-type heavy chain deposition disease/light and heavy chain deposition disease), monoclonal protein consists of the entire Ig and is detected only in glomeruli by immunofluorescence. Because the light and immunofluorescence microscopic appearances resemble immune complex glomerulonephritis, if immunoglobulin subgroups are not examined by immunofluorescence routinely, monoclonal accumulation may be missed by the pathologist. The possibility of detection of the circulating monoclonal IgG3 is low compared to IgG1/IgG2 and that is another challenge in diagnosis [33,34,35,45,46,47,48].

C3 glomerulopathy with monoclonal gammopathy is a different entity among the other MGRS lesions. Although the manifestation of the disease is glomerulonephritis, there are not any Ig deposits in the kidneys. C3 glomerulopathy is characterized only by C3 deposits in the glomeruli by immunofluorescence and it can be divided into two main groups according to the ultrastructure of the deposit under an electron microscope. One of them is C3 glomerulonephritis and it is characterized by subendothelial/subepithelial or mesangial granular deposits upon electron microscopy. The other is dense deposit disease and it is characterized by “sausage-like” high-density deposits in GBM [49,50]. The dysregulation of the alternative complement pathway is the main problem in both diseases. Some patients with C3 glomerulopathy have plasma cell dyscrasia and circulating monoclonal Ig. There are several hypotheses about the relationship between C3 glomerulopathy and monoclonal gammopathy [36]. This topic is not the subject of this article; however, it is important to emphasize that patients with the diagnosis of C3 glomerulopathy proven by biopsy should be investigated by the clinician in terms of monoclonal gammopathy (Figure 5).

Crystal-storing histiocytosis is a very rare disease associated with MM or MGRS. Although several sites can be affected, including the kidneys, perirenal adipose tissue, and the lungs, bone marrow involvement is most typical. Regardless of the site of involvement, the histiocytes with intralysosomal accumulation of Ig as crystals are determined by light microscope and most of the kappa LC cases are identified by DIF. We have no experience in the kidneys for crystal-storing histiocytosis [51,52].

## TREATMENT APPROACH

If the patient has a diagnosis of MM and has renal impairment, the treatment is more standard. The immediate start of antimyeloma therapy, dialysis (high cut-off hemodialysis), supportive care, high fluid intake, and avoidance of nephrotoxic agents are parts of standard of care. The value of plasma exchange is controversial; some studies showed encouraging results. These approaches may result in improvement of renal disease. Independence from dialysis is an important prognostic factor for survival. With the development of new agents such as thalidomide, bortezomib, and lenalidomide, the results are much better. Proteasome inhibitor (PI) (bortezomib)-based regimens are cornerstones in this setting. For eligible patients, autologous stem cell transplantation (ASCT) with 100-140 mg/m2 melphalan is feasible [7].

In settings without overt myeloma patients, the decision about the necessity of anti-myeloma therapy may be difficult. However, MGRS is associated with high morbidity due to renal disease and sometimes systemic findings may occur based on the monoclonal immunoglobulin. MGRS regroups all renal disorders caused by monoclonal proteins secreted by a nonmalignant B-cell clone (AL amyloidosis, cryoglobulinemia, MIDDs, Fanconi syndrome). In AL amyloidosis, the decision for myeloma-like therapy can be easily made, because the disease is usually systemic and has clinical features like a malignant disease rather than benign disorders. On the other hand, there are sufficient studies and data in this area, because it is a more common disorder, and standard care includes high-dose dexamethasone + melphalan or bortezomib-based regimens and ASCT in eligible patients [7].

In MIDDs, hematologic responses are best achieved with ASCT or PI-based therapies and are associated with improved renal outcomes [53]. Renal transplantation is feasible for MGRS, but to avoid recurrence after transplantation, control of the responsible B-cell clone is important [7,54]. However, it is not clear that small B-cell clones are truly curable; thus, the risk of disease recurrence cannot be eliminated totally.

Early diagnosis is important; if treatment is begun while renal functions are still preserved, long-term outcome results are better. MGRS is a heterogeneous and relatively rare entity and more collaborative studies and efforts of nephrologists and hematologists are required to improve its management.

## IMPORTANT ASPECTS

- Paraprotein can cause injury to the kidneys independent of its concentration.

- MGRS patients have a small B-cell clone and a low level of circulating paraprotein. Therefore, serum electrophoresis may not be sufficient and more detailed investigations may be required to detect monoclonal protein, like immunofixation and serum free LC assays, especially if the pathologist has any suspicion.

- The suppression of nephrotoxic monoclonal proteins in MGRS is very important for renal and also patient survival.

- Regular monitoring of the renal function, proteinuria, or hematuria can be very helpful for the early detection of the MGRS renal lesion with biopsy and the early effective treatment of cases with the diagnosis of monoclonal gammopathy with or without clinical importance for hematologists.

## Figures and Tables

**Schema 1:**
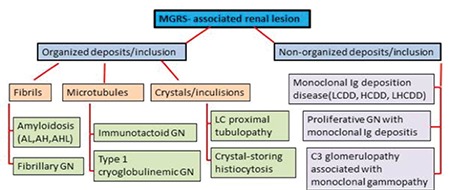
MGRS-associated renal lesions (modified from Bridoux et al. [36]). AH: Immunoglobulin heavy chain amyloidosis, AHL: immunoglobulin heavy and light chain amyloidosis, AL: immunoglobulin light chain amyloidosis, GN: glomerulonephritis, HCDD: heavy chain deposition disease, LCDD: light chain deposition disease, LHCDD: light and heavy chain deposition disease, MGRS: monoclonal gammopathy of renal significance.
